# Resisting the Resistance: Navigating BTK Mutations in Chronic Lymphocytic Leukemia (CLL)

**DOI:** 10.3390/genes14122182

**Published:** 2023-12-06

**Authors:** Alexandra Chirino, Skye Montoya, Anita Safronenka, Justin Taylor

**Affiliations:** Sylvester Comprehensive Cancer Center, University of Miami Miller School of Medicine, Miami, FL 33136, USA

**Keywords:** chronic lymphocytic leukemia, Bruton’s tyrosine kinase, resistance mutations, targeted therapy

## Abstract

Bruton’s tyrosine kinase (BTK) plays a key role in the B-cell receptor (BCR) signaling pathway and confers anti-apoptotic and proliferative properties to malignant B-cells in chronic lymphocytic leukemia (CLL). Small molecule BTK inhibitors were designed to bind BTK’s active site and block downstream signaling. These drugs have now been used in the treatment of thousands of patients with CLL, the most common form of leukemia in the western hemisphere. However, adverse effects of early generations of BTK inhibitors and resistance to treatment have led to the development of newer, more selective and non-covalent BTK inhibitors. As the use of these newer generation BTK inhibitors has increased, novel BTK resistance mutations have come to light. This review aims to discuss previously known and novel BTK mutations, their mechanisms of resistance, and their relationship with patient treatment. Also discussed here are future studies that are needed to investigate the underlying cause allowing these mutations to occur and how they incite resistance. New treatments on the horizon that attempt to maneuver around these resistance mutations can be met with new resistance mutations, creating an unmet need for patients with CLL. Novel therapies and combinations that address all forms of resistance are discussed.

## 1. Introduction 

Chronic lymphocytic leukemia (CLL) is characterized by the accumulation of B lymphocytes aberrantly expressing CD5+ in peripheral blood, bone marrow, and secondary lymphoid organs (lymph nodes and spleen) [1,2,3,4]. CLL is the most prevalent leukemia in western countries and originates from mature B-lymphocytes, though the exact cell of origin is still debated [1,2,5]. The prognosis for CLL can vary from being indolent to aggressive, with a transformation into diffuse large B-cell lymphoma (Richter syndrome) being the most feared outcome [6,7,8]. There is a higher susceptibility to CLL found in elderly and male patients; however, recent findings show that CLL diagnoses have expanded to encompass younger individuals, with nearly 15% of patients under the age of 55 [3,9,10,11]. This shift may result from increased blood screenings for a known family history, as a subset of CLL has a genetic predisposition, potentially leading to an earlier diagnosis of about 14 years [10,12,13,14]. Nonetheless, with few known risk factors, cases are sporadic, and the underlying pathogenesis of CLL is still being investigated. 

**Figure 1:**
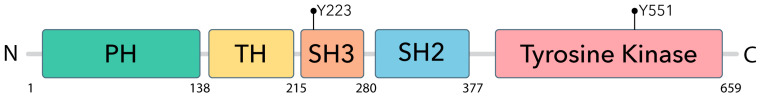
BTK Domains and activating tyrosinde phosphorylation sites. Map of the domaines found in BTK starting at the N terminal, BTK is made up of 5 different domains: the pleckstrin homology (PH), proline-rich TEC homology (TH), SRC homology 3 (SH3), SRC homology 2 (SH2), and a catalytic (kinase) domain ending with the C terminal, totalling 659 amino acids in length.

In CLL, the B-cell receptor (BCR) pathway is abnormally activated, leading to the hyperactivation of downstream protein kinases [5,15,16,17,18]. Bruton’s tyrosine kinase (BTK), a non-receptor Tec kinase early in the BCR pathway, has been shown to play a key role in B-cell development and proliferation [5,19]. BTK consists of five distinct domains: pleckstrin homology (PH), proline-rich TEC homology (TH), SRC homology 3 and 2 (SH3 and SH2), and a catalytic domain (kinase) [20]. The PH domain has a specific binding affinity for phosphatidylinositol 3,4,5-trisphosphate (PtdIns-3,4,5-P(3)), allowing it to respond to signaling mediated by phosphatidylinositol 3-kinase (PI3K) [4,21]. This responsiveness triggers the recruitment of proteins to the cell membrane, facilitating both protein localization and BTK translocation to the cell membrane. Point mutations in the PH domain perturb membrane binding and compromise its role in effective signaling [22]. The TH domain has a zinc-finger that plays a significant role in regulating BTK’s activity and stability though Zn^2+^ ion binding [4,21]. Both SH domains participate in crucial protein-protein interactions. The BTK SH2 domain, specifically, plays an essential role in the phosphorylation of phospholipase C-y2, triggering the mobilization of Ca^2+^ from the endoplasmic reticulum (ER) and leading to the anti-apoptotic and proliferative signaling of B cells [23,24]. The tyrosine kinase domain encompasses the tyrosine 551 (Y551) residue, which requires phosphorylation to initiate autophosphorylation at tyrosine 223 (Y223), located in the SH3 domain, and subsequent downstream signaling (Figure 1) [22,25]. 

Originally discovered in 1952 by Colonel Ogden Bruton, the importance of BTK in B-cell development is underscored by mutations found in X-linked agammaglobulinemia (XLA) [17,25]. Predominantly inherited by men, XLA is an immunodeficiency disorder that leads to a susceptibility to recurrent bacterial infections, due to a lack of antibodies [25]. Studies have shown BTK mutations are commonly found in those with XLA, where amino acid substitutions, splice defects, in-versions, termination, and insertions found in BTK can lead to a loss of function [26,27]. With disease severity depending on BTK enzymatic activity, even a frame shift changes such as hemizygous c.1632-1G>A can extremely lower the B-cell population and cause serious disease [26,27,28].

Considering the significant involvement of BTK and the impact it has on B-cells when dispossessed, scientists investigated the efficacy of inhibiting BTK in B-cell malignancies. As a cell signal blocker, these treatments attach to the ATP binding site of BTK, effectively preventing BTK from activating the BCR pathway [12,29,30]. Unable to proliferate, malignant b-cells stop growing and spreading, allowing for tumor reduction [19]. With this new development, inhibition of BTK shifted patient options from broader treatments (chemotherapy or radiation) to a more targeted approach (kinase inhibitors), revolutionizing the treatment landscape of CLL. Despite this success, prolonged exposure to BTK inhibitors (BTKi) can lead to mutational resistance. In this review, we will discuss the advances and challenges associated with targeting BTK in CLL, highlighting the potential implications for drug development and therapy selection for patients with CLL.

## 2. BTK Mutations Arise in Patients Treated with Covalent BTK Inhibitors

**Figure 2:**
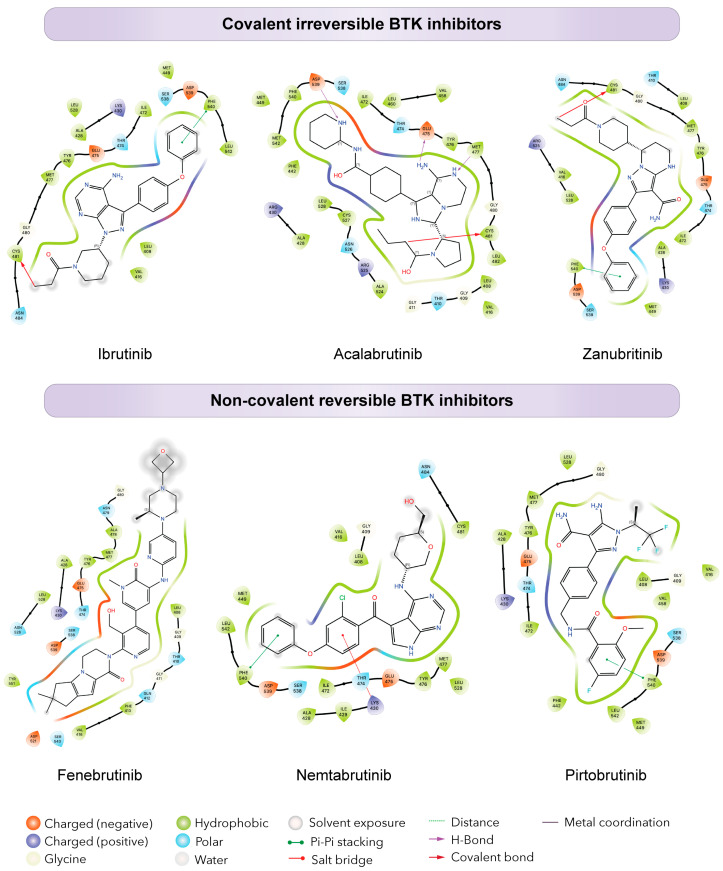
Differential binding of BTK inhibitors. Schematic diagrams showing protein-ligand interactions (Ligplots) showing the binding of covalent (**top**) and non-covalent (**bottom**) inhibitors in the ATP binding pocket of BTK. The top ligplots show binding to BTK of ibrutinib (PDB: 5P9J), acalabrutinib (PDB: 8FD9), and zanubrutinib (PDB: 6J6M). The bottom ligplots show binding to BTK of fenebrutinib (PDB: 5VFI), nemtabrutinib (PDB: 6E4F), and pirtobrutinib (PDB: 8FLL). Amino acid residues and corresponding three letter amino acid codes are shown in each plot, colored to specify amino acid types, charge, and interaction with the ligand.

The first-in-class covalent BTK inhibitor (cBTKi), ibrutinib, is an oral treatment that binds covalently and irreversibly to cysteine 481 (C481) in the ATP binding site of BTK (Figure 2) [12,31,32]. Ibrutinib was first was approved approved for the treatment of mantle cell lymphoma (MCL) in 2013, but soon after for CLL (2014), Waldenström’s macroglobulinemia (WM) (2015), and marginal zone lymphoma (MZL) (2017) [25,33,34,35]. This gave patients a new treatment option outside of the predominant use of chemoimmunotherapy and hematopoietic stem cell transplantation [12,32]. In addition to BTK, ibrutinib targets other signaling proteins such as HER2 and TEC family proteins, which can lead to undesirable off-target effects in patients [36,37]. Studies show that out of those who discontinue treatment, on average 30% had adverse effects (AE) including pneumonia, atrial fibrillation, and sepsis [38,39,40]. Depending on the study, up to 58% of patients stopped treatment after 2 years due to treatment intolerance, disease progression, or relapse [39,40,41].

After the recognition of ibrutinib toxicity due to off-target effects, second generation covalent inhibitors were developed to be more selective for BTK while still binding irreversibly (Figure 2) [42]. In 2019, acalabrutinib was approved by the Food and Drug Administration (FDA) for the treatment of CLL [43,44]. Similar in structure to ibrutinib, acalabrutinib also binds covalently to the C481 residue and inhibits the tyrosine phosphorylation of BTK and its subsequent signaling [29]. However, unlike ibrutinib, acalabrutinib was found to target fewer off-target kinases, causing fewer AEs [45,46]. Remarkably 72% of patients who discontinued ibrutinib treatment due to AEs did not experience these issues when re-challenged with acalabrutinib. Furthermore, only 12% stopped acalabrutinib treatment due to AE’s [45]. 

In 2023, zanubrutinib, a third covalent inhibitor gained FDA approval for CLL [47]. Like ibrutinib and acalabrutinib, it binds to the C481 residue of BTK. However, its design allows more specific binding with fewer off-target effects potentially lowering the number of AEs (Figure 2) [30,47]. In a recent update from the SEQUOIA study, results show that there were fewer AE’s and less discontinuation of treatment, with an impressive 82% progression-free survival (PFS) rate at 42 months [48]. Likely due to its structure, there are fewer cases of atrial fibrillation [30] making it safer for patients on clotting medications [49].

**Figure 3:**
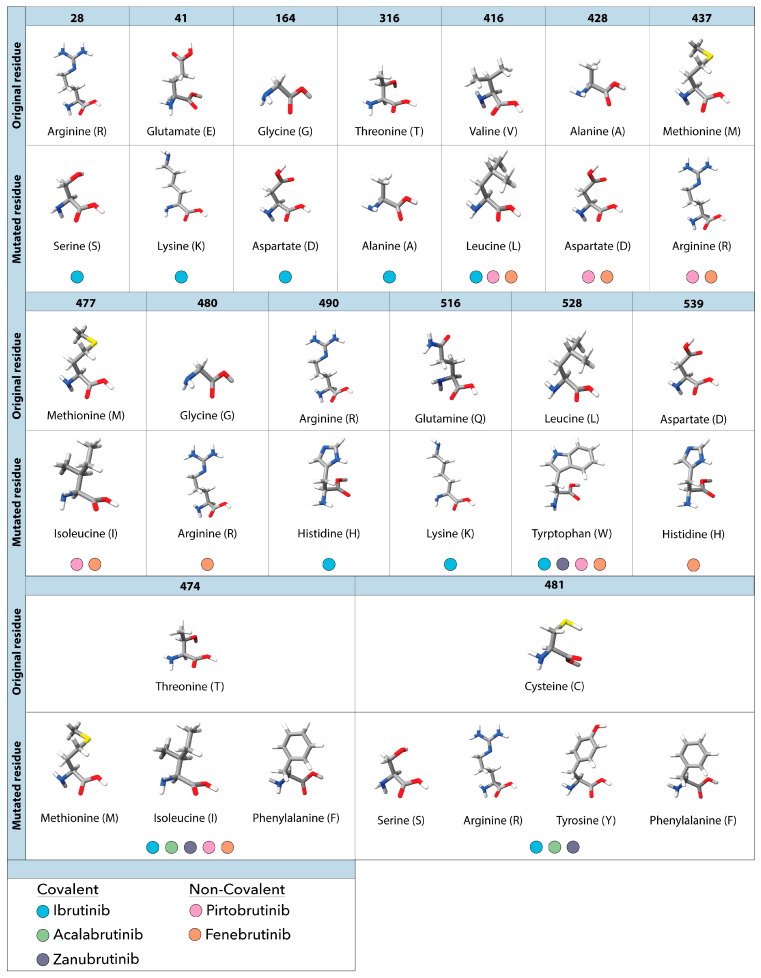
Observed amino acid mutations and their corresponding resistance to covalent and non-covalent BTK inhibitors observed in patients with CLL. The top portion of each row shows the original amino acid found at the specified residue in BTK. Directly below each original residue are the substituted mutated residues and the corresponding resistance to covalent (ibrutinib, acalabrutinib and zanubrutinib) and non-covalent (pirtobrutinib and fenebrutinib) BTK inhibitors. Each residue is colored to show specific elements such as hydrogen (white), oxygen (red), nitrogen (blue) and sulfur (yellow).

Despite the widespread success of ibrutinib these inhibitors, the emergence of mutational resistance in patients left many in need of alternative treatment options. Initial cases of resistance were observed in CLL patients undergoing ibrutinib treatment [40,50], with notable mutations emerging as early as 9 months after initiating treatment [51,52,53,54]. Sequencing results showed multiple mutations present in the cysteine 481 position, where the amino acid substitution from cysteine to serine (C481S), phenylalanine (C481F), threonine (C481T), tyrosine (C481Y), arginine (C481R) or glycine (C481G) blocked the covalent interaction between ibrutinib and BTK [55,56] (Figure 3). 

Studies on inhibitor resistance highlight the inability of covalent BTK inhibitors to covalently bind to and block phosphorylation, yet C481S and C481T form interactions similar to the original cysteine allowing them to retain kinase functionality [57,58,59,60]. This, in part, might explain why the C481S mutation is so common. Another variant, C481G, displays limited catalytic activity due to the absence of significant polar side chains. Nonetheless, it still permits a certain degree of ATP binding [42,61]. In contrast, substitutions like arginine, phenylalanine, tryptophan, and tyrosine prevent BTK Y223 autophosphorylation due to steric hindrance and structural clashing within the ATP binding pocket [42,62,63,64]. This causes mutations C481F/R/Y to be considered kinase dead. These mutants prevent ATP binding and BTK Y223 autophosphorylation (BTK activation) yet maintain downstream signaling, the mechanisms of which are still being investigated. It is worth noting that due to the similar binding motif of these cBTKis, the acquired resistance and disease progression due to the C481 mutations is a challenge that overlaps in all three [56,65,66,67].

As various studies have delved into covalent BTKi resistance, non-C481 mutations have become more prevalent. In one study, samples were obtained both before and during treatment with ibrutinib. The results of their investigation unveiled a range of BTK mutations extending beyond the widely recognized C481 mutations, including R28S, G164D, R490H, and Q516L (Figure 3) [68]. In another study, sequencing of 87 patients that were refractory to ibrutinib identified a subset of non-C481 mutations including E41K, T316A, V416L, T474I, and T474F, which comprised 1.3% of all BTK mutations (5 patients) (Figure 3) [69]. Notably, two of these patients concurrently carried the C481S mutation. Within another group of 20 patients, 8 individuals exhibited resistance to ibrutinib. Among 2 of the 8 patients, one acquired Q156L and R490H mutations in BTK, while the other acquired R28S and G164D mutations (Figure 3). Within the remaining 6 patients who developed resistance, all acquired the C481S mutation following treatment ranging from 17 to 40 months [54]. Similar non-C481 mutations have been observed in other cBTKis [54,68,70]. 

In an early study of patients treated with zanubrutinib, 10/13 patients had a C481 mutation, 7 of which has the Leu528Trp (L528W) mutation as well [64,65] (Figure 3). In a second study 4/8 patients had a C481 mutation detected, and 1/8 patients had both a C481 and L528W mutation [71]. BTK mutational resistance has been observed in patients treated with acalabrutinib as well. In a study conducted at Ohio State University, deep targeted sequencing was conducted on 16 patients that relapsed while on the treatment. Of these, 11 had BTK C481 mutations (63% C481S; 6% C481R and C481Y) and one had both a C481S mutation and a BTK T474I gatekeeper mutation [67] (Figure 3). These studies show that resistance to covalent inhibitors can occur even in newer generation BTK inhibitors made to be more selective with most being driven by C481 mutations. Thus, a need to develop non-covalent BTK inhibitors that do not require binding to C481 resulted in the next generation of BTK targeted therapies being non-covalent BTK inhibitors. 

## 3. Non-Covalent BTK Inhibitors in CLL and the Rise of Non-C481 Mutational Resistance

To overcome resistance and prevent disease progression, next generation non-covalent BTK inhibitors (ncBTKi) such as fenebrutinib, nemtabrutinib, and pirtobrutinib have been developed [72,73,74,75,76,77]. These inhibitors use hydrogen binding, ionic binding, and hydrophobic interactions to overcome mutational resistance from C481 mutations (Figure 2). This reversible binding could theoretically limit some of the selectivity of covalent BTKi’s; however, additional features of certain non-covalent BTKi’s also make them more selective. Each of these ncBTKis were designed to target both the wild type and C481 mutated BTK [78,79,80,81]. Nemtabrutinib forms hydrogen bonds to the E475 and Y476 residues of BTK. In addition to BTK, nemtabrutinib targets SRC kinases, AKT and ERK, [82,83] making it less selective when compared to other ncBTKis. Though no mutations have yet been described in patients taking nemtabrutinib, Wang et al have shown that cells expressing T474I and L528W are less sensitive to nemtabrutinib when compared to cells expressing wild-type (WT) BTK [84]. 

Fenebrutinib hydrogen bonds to the D539, M477, and K340 residues of BTK (Figure 2). Like nemtabrutinib, fenebrutinib showed less sensitivity in cells expressing T474I and L528W BTK mutations. Another study by Qi et al shows mutations L528S, G480R, and D539H create resistance to fenebrutinib in patient samples [85]. While fenebrutinib is highly selective for BTK, high doses can cause grade 3/4 AEs in patients which led to discontinuation of its development in CLL. Nonetheless, alternative uses for fenebrutinib in other diseases are still being tested [86,87].

Pirtobrutinib, the most recently developed ncBTKi, is a well-tolerated and highly selective BTKi. Pirtobrutinib has been approved by the FDA for MCL and is undergoing phase III trials for CLL (NCT05023980, NCT04666038). In addition to MCL and CLL, pirtobrutinib is also being tested for treatment use in a variety of malignancies, including WM (NCT05172700, NCT05734495, and NCT03740529), MZL (NCT04849416, NCT05990465, and NCT03740529), follicular lymphoma (FL) (NCT05990465), hairy cell leukemia (HCL), primary CNS lymphoma (NCT03740529), and diffuse large B-cell lymphoma (DLBCL) (NCT04849416 and NCT05990465) [24,88]. In CLL, in the phase I BRUIN trial, an overall response rate (ORR) of 82.2% was observed in 247 patients, with no obvious difference in response between those with wild-type BTK and those with a C481 mutation. Significantly, only 2.6% of patients discontinued treatment due to AE’s, with only 1.8% of patients having grade 3/4 AEs [89]. Pirtobrutinib is unique because it binds to the inactive form of BTK and does not require phosphorylation of Y551. Pirtobrutinib forms direct hydrogen bonds with residues E475 and M477 in the hinge region of BTK, establishes water-mediated hydrogen bonds with K430 and D539, and engages in pi-stacking interactions with F450 [90]. 

While the overall occurrence is low, studies following patients after prolonged treatment have begun to reveal mutations in BTK that cause resistance to pirtobrutinib allowing a disease progression [66,84,91]. Recently Wang et al. revealed the occurrence of 5 non-C481 mutations (V416L, A428D, M437R, T474I, and L528W) in patients initially responding to but ultimately relapsing on pirtobrutinib (Figure 3) [84]. These mutations cluster within the kinase domain of BTK [84] and result in sidechain point substitutions that impede the binding of both covalent and non-covalent BTK inhibitors [85,92]. Similar to the earlier described C481F/R/Y mutations, point mutations K430R, V416L, A428D, M437R, and L528W cause steric hindrance and structural clashing, preventing the binding of BTKis but also ATP, causing these mutations to be kinase deficient/dead. Even though ncBTKis don’t bind to the C481 residue of BTK, C481Y/F/R mutations are still observed in conjunction with other BTK resistance mutations in some patients [88,89,93,94]. In a recent clinical study (NCT03740529) researchers confirmed novel mutations can affect patient treatment outcomes. Pretreatment sequencing of patients enrolling on the pirtobrutinib study who previously received cBTKi treatment showed the common BTK C481S/R/Y and C481F mutations. Following the pirtobrutinib treatment, samples from patients who progressed were sequenced again and showed the clearance of C481S/Y/R/F mutations. Instead, new BTK mutations such as L528W, V416L, A428D, D539G, Y545N, and gatekeeper mutations like T474I/F/L/Y were detected (Figure 3) [92]. Future studies are necessary to determine which resistance mutations these ncBTKis can or cannot circumvent and their efficacy across a broader patient population.

## 4. Alternate Mechanisms Underlying Resistance to BTKis

**Figure 4:**
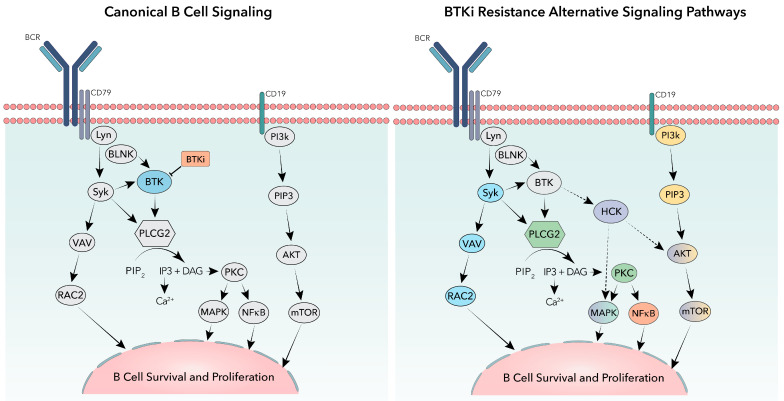
Canonical and alternative signaling pathways in B-cells. The canonical B-cell receptor signaling pathway is shown (**left**) highlighting BTK (blue) and BTK inhibitors (BTKi—Orange). BTKi resistance alternative signaling pathways are shown (**right**) highlighting alternative signaling from activating mutations in PLCG2 (green), mutations in NFkB (orange), signaling through activation of cell surface receptor CD19 (yellow), epigenetic signaling described from RAC2 activation (blue), or proposed noncatalytic signaling of kinase dead mutated BTK through HCK activation (purple).

Even when faced with potent signal inhibitors such as covalent and non-covalent BTKi’s, cancerous CLL cells can quickly adapt. One commonly occurring BTKi resistance mechanism arises from mutations in phospholipase C gamma 2 (*PLCG2*), the gene encoding for the direct downstream target of BTK PLCγ2. *PLCG2* mutations have been found in studies with both cBTKi and ncBTKi. The most commonly occurring *PLCG2* mutations are R665W, S707Y/F/P, 1139del, and D1140G/E/Y/N [52,56,69,77,95]. These mutations cause hypersensitivity to BTK and Syk phosphorylation leading to increased PLCγ2 activation (Figure 4) [64]. Though resistance mutations occur less frequently in *PLCG2* compared to *BTK*, *PLCG2* mutations are one of the most common non-BTK mutational resistance to BTKis.

Though the BCR signaling should be shut down once BTK is inhibited, previous studies have shown that these cancerous B-cells quickly adapt by using alternative signaling pathways. One example is through the activation of the cell surface receptor CD19, which leads to activation of PI3K and the AKT signaling pathway allowing CLL B-cells to survive and escape apoptosis (Figure 4) [96,97]. A small number of patients resistant to BTKis show no known BTK or PLCγ2 mutations (>20% on ibrutinib) as well as a lower number of cell apoptosis [98,99]. These cells may acquire other mutations in the BCR pathway, like NF-kB (Figure 4), to continue to proliferate independently of BTK, resulting in BTKi treatments becoming ineffective. 

Other studies address non-genetic methods of resistance. One study found that the primary resistance to BTKi is due to epigenetic changes that circumvent BTK inhibition. This study showed an epigenetic shift in activated B-cell DLBCL driven in part by the transcription factor TCF4 that enabled RAC2, a GTPase, to substitute for BTK in the activation of PLCγ2 (Figure 4) [100]. The authors also observed this resistance mechanism in CLL and suggested that epigenetic alterations may contribute to more BTKi resistance than currently thought.

Newer studies are beginning to describe a noncatalytic function of kinase dead mutant BTK [62,77,91,101]. In one study focused on BTK mutants C481F/Y/R and L528W, Yuan et al. use functional assays to show intact BCR signaling despite an absence of BTK kinase activity. They describe a dependency of these kinase dead mutants on toll-like receptor 9 (*TLR9*), unc-93 homolog B1 (*UNC93B1*), canopy FGF signaling regulator 3 (*CNPY3*) and hematopoietic cell kinase (*HCK*) [101]. In another recent study Dhami et al. found HCK was required in cells expressing the kinase dead BTK mutation C481F. Using the BTK C481F expressing cells with a deficient HCK mutation (K290E), Dhami et al. observed decreased activation of PLCγ2 as well as decreased calcium release following IgM stimulation, both of which are downstream responses to BTK activity [62]. To ensure that these results were of significance, gene editing was then used to knockout HCK in DT40 cells reconstituted with BTK WT or BTK C481F, and similar results were observed. Though the exact mechanisms are still being studied, this supports the idea that although the kinase function is suppressed by kinase dead BTK mutations, the presence of other BTK protein domains may act as a scaffold to allow alternative kinases, such as HCK (Figure 4), to be recruited and allow the continued downstream signaling and survival of malignant B-cells regardless of the treatment. 

## 5. Impact of BTK Mutations on Drug Development 

Due to the rising incidence of resistance to BTK inhibitors, alternate therapies designed to target non-BTK proteins have been developed. These have demonstrated effectiveness in treating both naïve and BTKi-refractory CLL patients. Downstream from the BCR, PI3Ks play a role in regulating cell proliferation. While there are three classes of PI3Ks, class one is particularly linked to the promotion of B and T cells. Inhibiting this class has demonstrated the induction of apoptosis in cancerous B-cells [102]. Among the four class one isoforms—α, β, delta, and γ—various inhibitors have been developed to prevent these kinases from activating the BCR pathway [103]. The targeting of PI3K seemed promising in therapeutic research. Specifically, Idelalisib, a PI3K inhibitor evaluated for CLL treatment (NCT00710528) demonstrated an overall response rate of 72% [104]. However, the excitement of these results was tempered by patient reports of severe (grade 3/4) side effects and a lack of complete responses [102]. In a study involving CLL patients, 42% had to cease using another such inhibitor because of its toxic effects, even after attempts to interrupt or modify the dosage [103]. Despite these inhibitors being designed for isoform selectivity, such reactions indicate that inhibiting the kinase might be inherently toxic [102]. This suggests that this treatment approach might be more suitable as a last-resort option for patients with progressive conditions.

Venetoclax, an inhibitor of B-cell lymphoma-2 (BCL2), has shown efficacy in patients by helping to overcome BTK mutational resistance and allowing up to 57% of patients to remain relapse free after 4 years [105,106]. Even so, this therapy faces its own challenges with protein mutations showing resistance up to 25 months prior to recurrence. Research indicates that nearly 50% of those who relapsed exhibited a mutation at the BCL2 binding site for venetoclax [106,107]. In cases where patients discontinued ibrutinib due to a relapse and initiated venetoclax as a sole therapy until further progression, Lucas and colleagues observed that 63% exhibited the C481S mutation both before and during the venetoclax treatment. Additionally, 45% acquired a *BCL2* mutation that was not present before the venetoclax treatment [108]. This reveals that there is an unmet need for patients with multiple BCR pathway mutations that result in a resistance to single-agent therapies. Preliminary data indicate that pirtobrutinib has a 58% overall response rate in those showing disease progression after being treated with multiple CLL therapies, including both cBTKis and venetoclax [109]. While comprehensive data on the impact of ncBTKi mutations on these patients is still required, the efficacy of pirtobrutinib for double-refractory patients remains clear.

As mutations lead to disease progression and fewer options, scientists have fought to overcome this scarcity and create new ways to help patients into remission. Combination therapies have sought to do this precisely. An ex vivo study found that CLL cells treated with ibrutinib were more sensitive to venetoclax and were primed for apoptotic dependencies due to the ibrutinib. Similar results have been shown in a phase 2 trial of acalabrutinib in combination with venetoclax suggesting that resistance may be prevented by inhibiting multiple targets simultaneously [106,110]. Understanding the potential of a more selective inhibitor paired with venetoclax, a phase 3 trial involving pirtobrutinib in combination with venetoclax is now in progress. With the primary objective being progression-free survival, there is optimism that the results will lead to improved patient outcomes [111]. 

Other studies of interest focus on approaches to degrade BTK rather than inhibit it. Two new chimeric targeting molecules (CTMs) from Nurix, NX-2127 and NX-5948, inhibit cell proliferation in lymphoma cells with BTK-C481S mutations and demonstrate superior tumor inhibition in xenograft models compared to ibrutinib [112,113]. These degraders have a bifunctional structure. One end of the molecule binds to the target protein (BTK), while the other end binds to an E3 ubiquitin ligase (cereblon). Bringing cereblon in proximity to BTK causes BTK to be polyubiquitinated and transported to the proteasome, a cellular machinery responsible for degrading unwanted or damaged proteins. The BTK degrader is then recycled for continued rounds of degradation. While clinical trials are currently being explored for CLL patients using NX-2127 and NX-5948, current ongoing data leans favorably with sustained BTK degradation for patients with B-cell malignancies [114]. As clinical evaluation for NX-2127 and NX-5948 progresses, there is hope for this type of treatment for CLL patients with challenging prognostic factors, including resistant BTK mutations [115]. This treatment provides options to those who have previously failed on BTK inhibitor therapies. 

HCK, another kinase integral to BCR signaling, is also worth investigating. Given that kinase-dead mutations rely on HCK for continued proliferation, targeting HCK could present another therapeutic avenue [62]. Using RK-20449, Dhami et al demonstrated the compound’s ability to selectively inhibit the proliferation of ibrutinib-resistant TMD8 cells, especially when compared to the parental TMD8 cells or TMD8 cells expressing the C481S mutation, which is in contrast to ibrutinib’s effects [62]. Although RK-20449 displayed limited selectivity, it still managed to suppress HCK phosphorylation in TMD8 ibrutinib-resistant cells that overexpressed the C481Y mutation [62]. Additionally, pre-clinical data from xenograft mouse studies using KIN-8194, another HCK inhibitor, indicated that mice implanted with TMD8 cells expressing the C481S mutation had a more favorable response to the inhibitor compared to other treatments, including ibrutinib. Although there was marked improvement in survival and significant tumor suppression, the disease still progressed in mice xenografted with BTK WT. Further research is essential to understand the potential efficacy and applicability of targeting HCK [116].

Lastly, the use of the chimeric antigen receptor T-cell (CAR-T) therapy has been tested as a treatment alternative. Using autologous T lymphocytes from CLL patients, these cells are re-infused after being transfected with a chimeric receptor recognizing CD19. Patients show an overall response rate of 57% [117]; however, the long term overall response rate was closer to 30% [117,118]. Current research suggests that combining CAR-T therapy with BTKi, or introducing new methods such as using NK effector cells, could potentially enhance remission rates as compared to using CAR-T therapy alone [117]. 

## 6. Conclusions

The important role of BTK inhibitors in the treatment of CLL has caused BTK to remains a focal point of both clinical and scientific development. This review highlights the increased occurrence of BTK mutations observed in patients and how these mutations can limit the efficacy of the approved covalent BTKi’s. Despite the development of non-covalent BTKi’s, novel BTK mutations have been observed to cause resistance and some of these mutations have the capability to cause cross-resistance to covalent BTK inhibitors. While studies discussed here underscore the progress that has been made, it is evident that these mutations persist and allow adaptive resistance mechanisms in CLL. Mutants in BTK can physically block inhibitor binding or allow BTK to cause resistance through noncatalytic functions such as the scaffolding mechanism. Future research efforts should aim to understand the full spectrum of BTK mutations and their implications in order to improve therapeutic development and ultimately patient outcomes. The dynamic interplay between the genomic landscape of CLL and targeted therapies like BTK inhibitors or degraders introduces both challenges and opportunities for better therapeutic options for patients.

## Data Availability

All data will be made available upon request.
